# Genome Sequence of *Actinomyces naeslundii* Strain ATCC 27039, Isolated from an Abdominal Wound Abscess

**DOI:** 10.1128/genomeA.01443-16

**Published:** 2016-12-29

**Authors:** Chiho Mashimo, Kazuyoshi Yamane, Takeshi Yamanaka, Hugo Maruyama, Pao-Li Wang, Satoshi Komasa, Joji Okazaki, Takayuki Nambu

**Affiliations:** a Department of Bacteriology, Osaka Dental University, Hirakata, Osaka, Japan; b Department of Removable Prosthodontics and Occlusion, Osaka Dental University, Hirakata, Osaka, Japan

## Abstract

Here, we present the complete genome sequence of *Actinomyces naeslundii* strain ATCC 27039, isolated from an abdominal wound abscess. This strain is genetically transformable and will thus provide valuable information related to its crucial role in oral multispecies biofilm development.

## GENOME ANNOUNCEMENT

It is well known that *Actinomyces* spp. are common bacteria in the normal flora of the mouth, and that they play an important role in the development of oral biofilm as one of the initial colonizers of tooth surfaces (1–3). *Actinomyces* spp. have been implicated in the dental caries process as acidogenic bacteria and could also be causative bacteria that make inroads to deeper tissues via traumatic wounds and surgical operations in both immunocompetent and immunocompromised individuals (4–6). Recently, the next-generation sequencing analysis of oral samples revealed that *Actinomyces* spp., especially *A. naeslundii* and *A. oris*, are core members of the healthy oral microbiome (7–12). Standard genetic engineering techniques are now applicable to *A. oris* (13, 14). On the other hand, there is no information regarding the gene modification system on *A. naeslundii*; therefore, we examined the transformability of several oral *A. naeslundii* strains and confirmed that strain ATCC 27039 was genetically tractable. The aim of the present study is to determine the full-genome sequence of* A. naeslundii* ATCC 27039.

Total bacterial DNA of strain ATCC 27039 was extracted from an overnight culture using a Nucleo spin tissue kit (Macherey-Nagel). A 20-kb SMRTbell library was prepared, and the genome was sequenced using the PacBio RS II system (Pacific Biosciences) on a single-molecule real-time (SMRT) cell using PacBio P6-C4 chemistry.

The* de novo* assembly of 153,093 reads with a mean length of 5,172 bp was completed using the hierarchical genome assembly process (HGAP) algorithm in SMRT Analysis software version 2.3 (15) and revealed a single contig approximately 3.04 Mb in length with an average coverage of 217.96×; the assembly was manually edited to circularize the overlapping ends of the genome. The final genome sequence is 3,040,449 bp in size and has a G+C content of 68.4%. The genome was then annotated using RAST version 2.0 (16), which successfully identified 3,229 coding sequences, as well as 60 RNA sequences. Of these, 43% of the annotated coding sequences fell within 318 subsystems available in the RAST database. The annotated data set presented here is expected to augment future study of this organism and provide resources for genetic manipulation.

### Accession number(s).

The genome sequence of *A. naeslundii* ATCC 27039 has been deposited in the DDBJ/EMBL/GenBank database under accession number AP017894.
